# *Pantoea dispersa* bacteremia in an immunocompetent patient: a case report and review of the literature

**DOI:** 10.1186/s13256-019-1969-z

**Published:** 2019-02-13

**Authors:** Nobuhiro Asai, Yusuke Koizumi, Atsuko Yamada, Daisuke Sakanashi, Hiroki Watanabe, Hideo Kato, Arufumi Shiota, Mao Hagihara, Hiroyuki Suematsu, Yuka Yamagishi, Hiroshige Mikamo

**Affiliations:** 1 Department of Clinical Infectious Diseases, Aichi Medical University Hospital, 〒480-1195 1-1 Yazakokarimata, Nagakute, Aichi Japan; 2 Department of Infection Control and Prevention, Aichi Medical University Hospital, Nagakute, Aichi Japan

**Keywords:** *Pantoea dispersa*, Bacteremia, Gram-negative rod, Cholangitis

## Abstract

**Background:**

*Pantoea* is a Gram-negative, non-encapsulated, non-spore-forming, ubiquitous straight rod which can be isolated from geographical and ecological sources such as plant surfaces, buckwheat seeds, human feces, and the environment. The genus *Pantoea* is a rare pathogen in a clinical setting, and is divided into 20 different species such as *Pantoea agglomerans*, *Pantoea ananatis*, *Pantoea deleyi*, *Pantoea dispersa*, *Pantoea septica*, *Pantoea stewartii* or *Pantoea rwandensis*. *Pantoea dispersa* has been reported to cause other infections, including respiratory infections, neonatal sepsis, and bloodstream infections. We report a case of *Pantoea dispersa* bacteremia caused by acute cholangitis. This is the first case report of *Pantoea dispersa* bacteremia caused by acute cholangitis as far as we had searched.

**Case presentation:**

A 38-year-old Japanese woman suffered from acute cholangitis; a blood culture showed that Gram-negative rod was positive. The treatment was successful with intravenously administered meropenem, and it was switched to orally administered levofloxacin according to microbiological susceptibility. The organism was identified as *Pantoea dispersa* by both genetic investigation by 16S ribosomal RNA and additional biochemical tests. To the best of our knowledge, this is the first case report of *Pantoea dispersa* bacteremia caused by acute cholangitis.

**Conclusion:**

The epidemiology and clinical features of *Pantoea dispersa* are still unknown. More cases of infections caused by *Pantoea dispersa* might be revealed with advancing technical methods, such as matrix-assisted laser desorption/ionization time-of-flight mass spectrometry or 16S ribosomal RNA analysis. Physicians must know that a variety of infections caused by *Pantoea dispersa* could occur in immunocompromised as well as immunocompetent patients.

## Background

*Pantoea* is a Gram-negative, non-encapsulated, non-spore-forming, ubiquitous straight rod which can be isolated from geographical and ecological sources such as plant surfaces, buckwheat seeds, human feces, and the environment [1, 2]. The genus *Pantoea* is a rare pathogen in a clinical setting, and is divided into 20 different species named *Pantoea eucalyptii*, *Pantoea agglomerans*, *Pantoea vagans*, *Pantoea conspicua*, *Pantoea deleyi*, *Pantoea anthophila*, *Pantoea brenneri*, *Pantoea ananatis*, *Pantoea allii*, *Pantoea stewartii*, *Pantoea cypripedii*, *Pantoea calida*, *Pantoea gavinae*, *Pantoea dispersa*, *Pantoea septica*, *Pantoea wallisii*, *Pantoea eucrina*, *Pantoea rodasii*, *Pantoea rwandensis*, and *Pectobacterium carotovorum* [2, 3]*. P. agglomerans* is the most prominent species in humans, formerly named *Enterobacter agglomerans*.

*P. dispersa* has been reported to cause other infections, including respiratory infection [4], neonatal sepsis [5], and bloodstream infection [6]. This microbe has been known to cause infections in immunocompromised patients but not in immunocompetent patients. Here we report a case of *P. dispersa* bacteremia caused by acute cholangitis. This is the first case report of *P. dispersa* bacteremia caused by acute cholangitis, as far as we could search.

## Case presentation

**Fig. 1:**
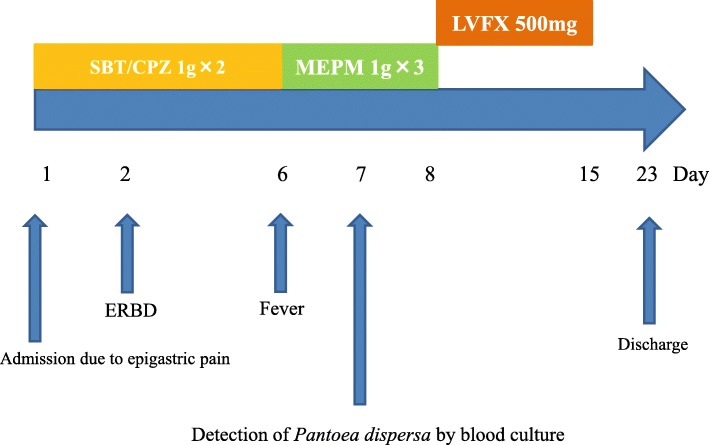
The clinical course of this case. *CPZ* cefoperazone, *ERBD* endoscopic retrograde biliary drainage, *LVFX* levofloxacin, *MEPM* meropenem, *SBT* sulbactam

**Fig. 2:**
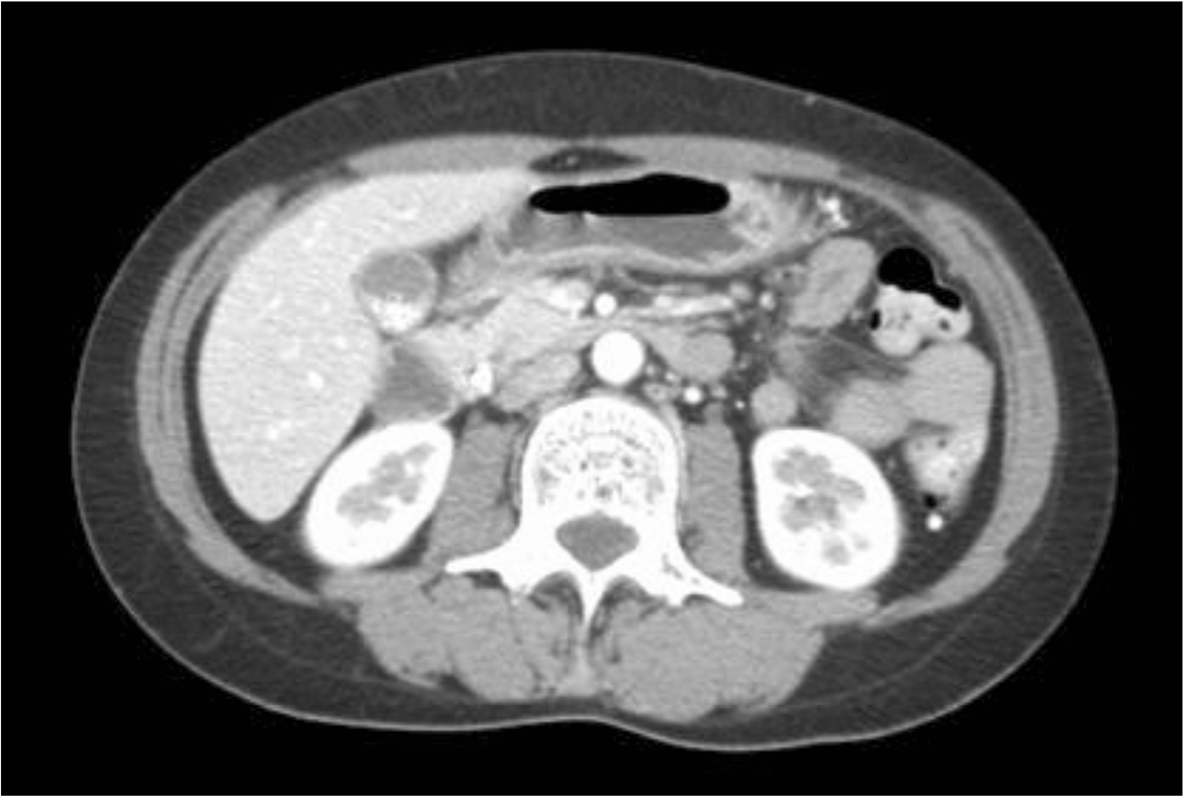
Abdominal computed tomography shows gallstones with gallbladder wall thickening

A 38-year-old Japanese woman came to our institute with a complaint of epigastric pain after meals. She had no medical history and no exposures to plants or animals prior to her hospital stay or invasive procedures. She never smoked tobacco and was not an alcohol consumer. She was diagnosed as having acute cholangitis induced by stone based on symptoms and laboratory findings (Table 1), and was admitted (Fig. 1). Her body temperature was 37.1 °C, blood pressure 97/57 mmHg, and heart rate 85/minute. She did not exhibit any jaundice. An abdominal examination revealed tenderness on the epigastric portion. No rebound tenderness was confirmed. Her cardiac, respiratory, and neurological examinations were normal. Abdominal computed tomography (CT) findings showed gallstones with gallbladder wall thickening (Fig. 2). Antibiotic therapy of sulbactam (SBT)/cefoperazone (CPZ) was started empirically at the same time. When undergoing endoscopic nasobiliary drainage, she had a high fever and two sets of blood cultures were obtained on day 6. Growth of Gram-negative rods was reported in both aerobic and anaerobic blood cultures within 24 hours on BACTEC™ (BD, Tokyo, Japan). Antibiotic therapy of meropenem (MEPM) was started empirically. Our patient’s clinical condition and laboratory data improved rapidly. After 3 days of intravenously administered MEPM, the antibiotic therapy was switched to orally administered levofloxacin (LVFX) 500 mg daily for another 7 days according to microbiological sensitivity. The infection did not recur and she was discharged on day 28. During 1 year, recurrence of the infection was not observed.

**Table 1:** Laboratory findings on admission

WBC	13,200/μL	CK	36 IU/L
Neu	92.4%	γ-GTP	129 IU/L
Lym	5.3%	Amy	61 IU/L
Mono	1.9%	TP	7.3 g/dL
Eos	0.1%	Alb	4.2 g/dL
RBC	410 × 10^4^/μL	BUN	9.6 mg/dL
Hb	11.7 g/dL	Cre	0.6 mg/dL
Plt	19.2 × 10^4^/μL	Na	136 mmol/L
AST	30 IU/L	K	4.1 mmol/L
ALT	63 IU/L	Cl	98 mmol/L
T-Bil	1.1 mg/dL	Ca	9.1 mg/dL
LDH	180 IU/L	Glu	66 mg/dL
ALP	341 IU/L	CRP	17.7 mg/dL

*Alb* albumin, *ALP* alkaline phosphatase, *ALT* alanine aminotransferase, *Amy* amylase, *AST* aspartate aminotransferase, *BUN* blood urea nitrogen, *Ca* calcium, *CK* creatine kinase, *Cl* chlorine, *Cre* creatinine, *CRP* C-reactive protein, *Eos* eosinophil, *γ-GTP* γ-glutamyltransferase, *Glu* glucose, *Hb* hemoglobin, *K* potassium, *LDH* lactate dehydrogenase, *Lym* lymphocyte, *Mono* monocyte, *Na* sodium, *Neu* neutrophil, *Plt* platelet, *RBC* red blood cell count, *T-Bil* total bilirubin, *TP* total protein, *WBC* white blood cell count

First, the pathogen by positive blood culture was identified as *Klebsiella ozaenae* by means of a MALDI Biotyper® (Bruker Daltonics). Subsequently, genetic investigation by 16S ribosomal RNA (rRNA) analysis was performed in order to identify this organism. Finally, the pathogen was identified as *P. dispersa* with 100% homology (1343 of 1343 bases) on the EZ taxonomy database (http://www.ezbiocloud.net/eztaxon). We also conducted additional biochemical tests using API® 50 CH kit, according to previous reports to confirm the isolate as *P. dispersa*. The organism had no activities of esculin and salicin, and had activities of lactose, melibiose, and gentiobiose, which were consistent with *P. dispersa* [6].

Antimicrobial susceptibility testing was performed according to Clinical and Laboratory Standards Institute (CLSI) criteria for *Enterobacteriaceae* [7] using the newly developed, fully automated microbiology system, RAISUS (Nissui Pharmaceuticals Co., Ltd., Tokyo). The organism was susceptible to all antimicrobial agents tested, including ampicillin, cefazolin, gentamicin, LVFX, and trimethoprim-sulfamethoxazole (Table 2).

**Table 2:** Antimicrobial susceptibility of *Pantoea dispersa* isolated from blood culture

Antimicrobial agents	MIC (μg/mL)	Interpretation
Ampicillin	≤ 8	S
Minocycline	≤ 4	S
Amikacin	≤ 4	S
Aztreonam	≤ 1	S
Ceftazidime	≤ 1	S
Cefazolin	8	R
Cefepime	≤ 2	S
Cefmetazole	≤ 16	S
Ciprofloxacin	≤ 0.063	S
Cefotiam	≤ 2	
Cefotaxime	≤ 1	S
Fosfomycin	≤ 64	
Imipenem	≤ 0.5	S
Levofloxacin	≤ 0.125	S
Piperacillin	≤ 16	S
Trimethoprim/sulfamethoxazole	≤ 2/38	S
Meropenem	≤ 0.125	S
Tazobactam/piperacillin	≤ 4/4	S

*MIC* minimum inhibitory concentration, *R* resistant, *S* susceptible

## Discussion

*Pantoea* is a genus of Gram-negative bacteria of the family *Enterobacteriaceae* that was recently separated from the *Enterobacter* genus. They have also recently been shown to cause infections in humans [1–6]. However, only a limited number of clinical cases with bacteria belonging to this genus have been described. Thus, there is not enough information on its pathogenic mechanism.

A total of five cases of infections by *P. dispersa* including ours have previously been reported as shown in Table 3. Two of the five cases were neonates, and the other three cases were adults. The sites of infections varied such as respiratory or blood stream infections. As for the underlying diseases of the three adults, one patient with leukemia was immunocompromised and the other two were immunocompetent. In terms of the outcomes, all patients were improved. Epidemiology and clinical features of *P. dispersa* infection are still unknown due to its rarity and the difficulty in accurate identification. A previous report documented that more than 10% of clinical isolates of *P. agglomerans* were misidentified as species of the genus *Enterobacter* by the VITEK® MS system [8]. In the present case, the isolate was initially misidentified as *Klebsiella ozaenae* by MALDI Biotyper®. Finally, 16S rRNA analysis confirmed that the isolate was *P. dispersa*. More cases could be missed due to misidentifications as *P. dispersa*. A variety of infections caused by *P. dispersa* have been reported [4–6]. More cases of infections caused by *P. dispersa* might be revealed with advancing technical methods, such as matrix-assisted laser desorption/ionization time-of-flight mass spectrometry (MALDI-TOF-MS) or 16S rRNA analysis.

**Table 3:** Previous reports of *Pantoea dispersa* infections

Author (year)	Sex	Age	Site of infection	Underlying disease	Treatment	Outcome
Schmid *et al*. (2003) [4]	F	71	Respiratory infection	Acute myeloid leukemia	CAM AMPC/CVA	Improved
Hagiya and Otsuka (2014) [6]	M	64	Bloodstream infection Bacteremia	DCM DM SSS	Cefepime	Improved
Mehar *et al*. (2013) [5]	ND	Neonate	Sepsis	Not described	TAZ/PIPC AMK	Improved
Mehar *et al*. (2013) [5]	ND	Neonate	Sepsis	Not described	SBT/ABPC AMK	Improved
Current case	F	38	Bacteremia Cholangitis	Choledocholithiasis	Meropenem	Improved

*AMK* amikacin, *AMPC/CVA* amoxicillin/clavulanate, *CAM* clarithromycin, *DCM* dilated cardiomyopathy, *DM* diabetes mellitus, *F* female, *M* male, *ND* not described, *SBT/ABPC* sulbactam/ampicillin, *SSS* sick sinus syndrome, *TAZ/PIPC* tazobactam/piperacillin

All species of the genus *Pantoea* can be *isolated* from feculent material, plants, and soil [2]. However, our patient had no contact with these sources. The isolate was susceptible to amikacin, cefepime, cefotaxime, ciprofloxacin, MEPM, and aztreonam, and resistant to cefazolin. Fortunately, our patient survived because appropriate antibiotic therapy was rapidly started. These results were similar to those of previous reports [4–6]. Of note, *P. dispersa* bacteremia can occur not only in immunocompromised hosts but also in immunocompetent patients. Although all cases improved, the pathogenic and clinical importance of *P. dispersa* infection are unclear. Additional case reports of *P. dispersa* infections could help physicians understand the pathogenetic potential of this organism.

## Conclusion

We experienced a case of *P. dispersa* bacteremia caused by acute cholangitis, which is the first report as far as we could search. Although *P. dispersa* could cause a variety of infections in immunocompromised as well as immunocompetent patients, some cases of *P. dispersa* infections might be misdiagnosed as other pathogens infection. More cases of infections by *P. dispersa* should be collected and examined to clarify the epidemiology of *P. dispersa* infections.

## Abbreviations

CLSI: Clinical and Laboratory Standards Institute
CPZ: Cefoperazone
CT: Computed tomography
LVFX: Levofloxacin
MALDI-TOF-MS: Matrix-assisted laser desorption/ionization time-of-flight mass spectrometry
MEPM: Meropenem
rRNA: Ribosomal RNA
SBT: Sulbactam
